# The Influence of the COVID-19 Pandemic on Mental Well-Being and Psychological Distress: Impact Upon a Single Country

**DOI:** 10.3389/fpsyt.2020.594115

**Published:** 2020-11-11

**Authors:** Nicola S. Gray, Chris O'Connor, James Knowles, Jennifer Pink, Nicola J. Simkiss, Stuart D. Williams, Robert J. Snowden

**Affiliations:** ^1^Department of Psychology, Swansea University, Swansea, & Swansea Bay University Health Board, Swansea, United Kingdom; ^2^Aneurin Bevan University Health Board, Newport, United Kingdom; ^3^Department of Psychology, Swansea University, Swansea, United Kingdom; ^4^Independent Marketing, Swansea, United Kingdom; ^5^School of Psychology, Cardiff University, Cardiff, United Kingdom

**Keywords:** mental health, psychological well-being, psychological distress, COVID-19, mental disorder, K10, Warwick Edinburgh Mental Well-being Scale (WEMWBS)

## Abstract

The COVID-19 pandemic is likely to have affected the psychological well-being and mental health of many people. Data on prevalence rates of mental health problems are needed for mental health service planning. Psychological well-being and prevalence of clinically significant mental distress were measured in a large sample from Wales 11–16 weeks into lockdown and compared to population-based data collected in 2019 before the COVID-19 pandemic. Data were collected using an online survey disseminated across Wales and open to adults (age 16+) from 9th June to 13th July 2020. Psychological well-being was indexed *via* the Warwick-Edinburgh Mental Well-being Scale, and psychological distress was indexed *via* the K10. Data from 12,989 people who took part in this study were compared to that from April 2018 - March 2019, gathered by the National Survey for Wales (*N* = 11,922). Well-being showed a large decrease from 2019 levels. Clinically significant psychological distress was found in around 50% of the population (men = 47.4%, women = 58.6%), with around 20% showing “severe” effects (men = 17.0%, women = 20.9%): a 3–4-fold increase in prevalence. Most affected were young people, women, and those in deprived areas. By June-July 2020 the COVID-19 pandemic had dramatic effects on the mental health of people living in Wales (and by implication those in the UK and beyond). The effects are larger than previous reports. This probably reflects that the current data were taken deeper into the lockdown period than previous evaluations. Mental health services need to prepare for this wave of mental health problems with an emphasis on younger adults, women, and in areas of greater deprivation.

## Introduction

The COVID-19 pandemic has caused widespread problems across the world that are likely to have adverse effects on mental health and well-being (1, 2). The problems are multifarious and include fear of one's own illness or death, fear of illness or death of a loved one, fears due to loss of employment, and the effects of social and physical isolation in response to the pandemic (3).

Early reports showed that care-workers suffered from high rates of depression and anxiety during the first few weeks of the initial outbreak in China, with women being particularly affected (4). However, as Perlis (5) notes, this leaves open many questions, such as whether these rates are due to being a health-care worker, simply living in the midst of such an outbreak, or due to the possible consequences of quarantine or other restrictions. Perlis (5) also raises the issue of whether these symptoms will persist or even worsen over time.

There have now been several reports on the mental health of specific populations during the COVID-19 pandemic. McGinty et al. (6) sampled over 1,000 individuals from the USA in a single week in April 2020 and compared this to a national sample taken during 2018. Using the K6 (7) measure of psychological distress, they noted that 13.6% reported “serious” levels of psychological distress during the pandemic period compared to 3.9% in 2018. These levels were moderated by age and income, with 18–29 year olds having a prevalence of 24.0% and those with the lowest income having a prevalence of 19.3%. Pierce et al. (8) studied 17,000 individuals across the UK in a single week in April 2020 (1 month into the COVID-19 lockdown) and compared this with previous data. Using the GHQ-12 (9), a measure of mental health relative to the person's usual mental state, they found a modest increase in GHQ-12 scores that corresponded to an increase in psychological distress from 18.9% pre-COVID-19 to 27.3%. These increases were greater in the younger age groups, and for women. This pattern of results has been replicated by other studies that occurred in the early phase of the pandemic [e.g., (10–12)] and have been extended to show high levels of thoughts of self-harm and suicide in the first month of the lockdown in the UK (13) with, again, a higher incidence rate for women.

There are also an increasing number of yet-to-be peer-reviewed reports that attest to deterioration of mental health due to the COVID-19 pandemic (14–24). However, the current report does not provide an in-depth review of this material due to concern expressed by others (25, 26) that some of these data may be misleading due to not having been appropriately peer-reviewed.

The present study examined psychological well-being and mental distress in the population of Wales during the period of lockdown, and took measures of key demographic variables that might moderate these effects. The study adds to previous studies in several ways. First, data was taken for both psychological well-being and psychological distress. These concepts are distinct, but correlated, and are not merely the inverse of each other. Well-being represents feelings of happiness and a sense of purpose which can remain even in the presence of mental illness, distress, or suffering (27–29). So far, there have been no studies examining the effects of the COVID-19 pandemic on mental well-being.

Second, the present study examined a period deeper into the pandemic. For instance, most studies (4, 6, 8, 10–12) gathered data within the first few weeks of the pandemic, whereas the data for the present study were gathered 11–16 weeks into the lockdown period. It is possible that psychological well-being will be more severely impacted after a prolonged exposure to pandemic related stressors. For example, Kato et al. (30) argued that longer lasting social isolation increases loneliness and that loneliness is, in turn, a crucial risk factor for a number of forms of mental health difficulty, including anxiety, depression and addiction disorders. Alternatively, it is possible that people will learn and adjust to the situation over time and that the psychological stress caused by the pandemic diminishes over time—see Perlis (5). This is an empirical argument and it is important to evaluate the strength of these effects.

Third, studies such as Pierce et al. (8) used a measure of mental health that uses the person's usual mental health as a baseline. Hence, a person who is only mildly distressed relative to their normal healthy state will score higher on the GHQ-12 than someone who remains in a chronic state of severe distress or mental illness. While the GHQ-12 excels in examining changes in mental health, it is less able to gauge the absolute levels of well-being or mental health in the population. The K10 (7) is better placed to do this as it asks for frequency of symptoms and is designed to classify individuals into categories of psychological distress (none, mild, moderate, or severe).

The first COVID-19 case was confirmed in Wales on the 28th February 2020 with the first death reported on the 16th March 2020. By 20th March 2020 all mainstream schools across Wales were closed. On March 23rd 2020 the UK Government issued a lockdown of the UK and only essential services remained open. Gatherings of two or more people (except for individuals in the same household) were banned, whilst pubs, restaurants, and shops selling “non-essential goods” were ordered to close. Individuals in Wales were informed they could no longer travel more than 5 miles from their home unless necessary.

At the start of the present survey (9th June 2020), the UK had the second highest number of cumulative deaths in the world, only surpassed by the USA (31). At this point, over 9% of all reported deaths resulting from COVID-19 had occurred in the UK. Of a total 39,277 deaths, 1,435 deaths had occurred in Wales, a rate of 45.5 deaths per 100,000 people (32, 33). During the period of the survey, reported deaths from COVID-19 continued to increase. On the 6th July 2020 lockdown restrictions began to ease in Wales so that people were now allowed to travel more than 5 miles, although the other restrictions remained in place.

By the end of the survey (13th July 2020), the UK had the third highest death toll from the pandemic (30), having reported another 1,711 deaths during this period. During this period, the mortality rate increased further in Wales, to 49.0 deaths per 100,000 people (31, 32). Restrictions were eased further on the 31st July 2020. Pubs and restaurants were able to open indoor areas on 3rd August 2020. Up to 30 could meet outdoors, and children under 11 would no longer have to socially distance. Swimming pools, gyms, leisure centers and indoor play areas were allowed to reopen from 10th August 2020, but all with social distancing measures in place.

### Objectives

The main objective of the study was to measures the psychological well-being of the Welsh population during a period 11–16 weeks into the period of lockdown due to the COVID-19 sample and compare this to levels in a period before the lockdown. In addition, it aimed to examine the prevalence of significant levels of psychological distress during the COVID-19 lockdown. In addition, the study looked at factors that might mitigate or aggravate such distress. We hypothesized that psychological well-being would be reduced due to COVID-19 and that this effect would be greater in women than in men, in those of a younger age, and in those people living in areas of high deprivation (8). In line with this reduction in psychological well-being, we hypothesized that levels of mental distress would be high, with the same demographic factors aggravating these levels of distress (6, 8, 10–12).

## Materials and Methods

The study was approved by the Research Ethics Committee at the College of Health and Human Sciences, Swansea University. The project is registered with ISRCTN ref: 21598625. The study protocol is published at: https://www.swansea.ac.uk/psychology/research-at-the-department-of-psychology/research-protocols/.

With respect to mental health, it is important to compare the situation during the lockdown period to data from before this period in order to gauge the effects of the pandemic. The National Survey for Wales (NSfW) performs regular surveys of the Welsh population and had data on mental well-being from 11,922 respondents during the period April 2018 to March 2019 (34). We will term this as the “2019 sample.” Therefore, the present study (which we will term the “2020 sample”) used the same measure of mental well-being, the Warwick-Edinburgh Mental Well-being scale [WEMWBS (28)], in order to be able to compare the 2020 sample to this 2019 sample.

### Participants

Participants for the 2020 sample were recruited *via* online snowball sampling. The survey was advertised *via* a programme of social media advertisements and emails designed to cover the population of Wales. This included emails and tweets being sent to organizations across Wales asking them to publicize the existence of the survey to their staff and service-users and giving the URL of the survey website to be able to access the survey. Many organizations agreed to support the research and to advertise and disseminate the survey (see Acknowledgments). This included all seven Health Boards in Wales; the four police forces in Wales; the Welsh Ambulance Service Trust; the Fire & Rescue Service; many large employers across Wales, including large government organizations; care homes for elderly residents; homelessness organizations; GPs; the Welsh Farmers' Union; and third sector partnership organizations (e.g., charitable organizations supporting specific sectors of the community). The survey was also advertised *via* newspapers, radio programmes, and celebrity tweets.

In order to match the 2020 sample to the 2019 sample (34), the 2020 sample recruited a minimum number (*n* = 250) from each of the 22 Local Authorities across Wales. This also ensured good coverage of all seven Health Boards across Wales.

Data for the 2019 sample were taken from the National Survey for Wales (NSfW) conducted between April 2018 to March 2019 (34). This is a large-scale survey of adults in Wales run by the Office for National Statistics on behalf of the Welsh Government. Twenty thousand participant households in Wales were chosen at random from the Royal Mail's publicly available address list and were invited to take part. Face to face interviews were conducted on 11,922 participants. Information was collected on several topics including population health and well-being, children and education and social care services. The survey aimed to gather an understanding of life across Wales, and the results are used by the Welsh Government to assist in policy and decision making and directing resources to where they are needed the most.

### Procedures

The survey was open from 9 June 2020 to 13 July 2020. The survey was administered online (Qualtrics software, Version June 2020, Provo, UT, USA, Copyright © 2020Version) for the vast majority of participants (>99%), and was available in both English and Welsh language versions.

The survey also had a dedicated telephone line that was widely advertised so hard to reach sectors of the population without access to the internet or electronic devices could request a paper-based survey (with stamped addressed envelope) and thus were able to engage with the survey. The survey was designed to take around 10 min to complete (see Results).

### Measures

The survey comprised various sections. The first section was an information sheet and informed consent form. The next section asked demographic questions that included gender, age group, ethnicity, occupation, and postcode (used to calculate the deprivation index) among others. The next section covered the person's current thoughts and feelings and included both the WEMWBS and the K10 (see below). The next section looked at the person's current stressors and their resilience to stress in order to examine what aspects of the pandemic were related to poor psychological well-being and whether there were personal factors that might mitigate against poor psychological well-being. The final section examined if there were aspects of the lockdown that people had enjoyed during the pandemic (e.g., spending more time with their family), in order to examine if there are positive factors that mitigate against poor psychological well-being due to the pandemic. Given the large dataset generated, data from these final two sections were not analyzed here and so the details are not provided. We hope to disseminate these data at a later date.

In accordance with recent ethical considerations for mental health research during the COVID-19 pandemic (35), participants were informed that the study would ask questions about their emotional well-being before they were asked to provide fully informed consent. Further, as suggested by Townsend et al. (35) there was a section at the end of the survey that attempted to mitigate any distress caused by the survey. This section asked participants to consider whether there were any aspects of the COVID-19 pandemic that they had enjoyed (e.g., “spending more time with one's family” or “enjoying a renewed sense of community spirit”). At the end of the study, participants were also provided with a debrief form that thanked them for their important role in the research and then signposted to three separate services, available across Wales, that offered free, 24/7, confidential listening and support *via* the telephone, SMS messaging or e-mail. Participants were encouraged to contact the provided services if they were experiencing any current emotional difficulties.

#### Mental Well-Being

Current mental well-being (over the past 2 weeks) was assessed *via* the Warwick-Edinburgh Mental Well-being Scale [WEMWBS (28)]. The WEMWBS has been used in studies across the world [e.g., (36)]. It has strong positive relationships to other measures of positive mental health (28, 37). However, it has a more modest negative relationship to measures of mental ill-health (e.g., GHQ-12) suggesting that the two concepts are not merely the inverse of each other (27, 28).

The WEMWBS contained 14 items covering issues such as positive affect, level of functioning, and relationships over the past 2 weeks. Items are answered on a five-point Likert scale with respect to frequency (from “*none of the time*” to “*all of the time*”) to give a score ranging from 14 to 70, with greater scores indicating greater well-being. The internal consistency of the WEMWBS was high in the 2020 sample (Cronbach α = 0.94).

#### Psychological Distress

Current level of psychological distress was assessed by the Kessler Distress Scale [K10: (7)]. The K10 has been used in studies across the world (35, 38, 39) and is available in several languages [e.g., (40, 41)]. It has good ability to predict serious mental illnesses in the general population (41–43).

The K10 contains 10 items measuring current psychological distress, and, in particular, symptoms of anxiety and depression. Items are rated on a five-point Likert scale with respect to frequency (from “*none of the time*” to “*all of the time*”) to give a score from 10 to 50, with greater scores indicating greater levels of psychological distress. The standard K10 asks people to rate their distress over the past 30 days. However, this was amended to cover the past 2 weeks to match the time period of the WEMWBS. The internal consistency of the K10 was high in the 2020 sample (Cronbach α = 0.93).

#### Welsh Index of Multiple Deprivation

The Welsh Index of Multiple Deprivation (WIMD) is produced by the Welsh Government (44) and is a measure of relative deprivation for 1909 areas of Wales (1 = most deprived, 1,909 = least deprived) each containing an average of 1,600 people. It assesses deprivation as “*the lack of access to opportunities and resources which we might expect in our society*” (44). It also has an interactive tool that allows for a postcode to be translated into the WIMD rank.

## Results

### Demographics

In total, 15,469 people started the survey. Of these, 2,417 did not complete over 50% of the survey (this corresponds to not having completed the WEMWBS, which was the primary outcome measure) and were excluded from further analysis. We do not have any information on the reason(s) behind these individuals not completing the survey.

Analysis of the time taken to complete the survey found that the median time was 647 s (IQR: 510–863) and people (*n* = 63) who had taken <240 s were removed as we judged that such fast completion was not commensurate with carefully answering the questions. Hence, data from 12,989 people are reported, although not all people completed all sections or all questions. Numbers of people involved in each analysis are stated in the appropriate place.

Demographic data from the 2020 sample are displayed in Table 1, alongside data from the 2019 sample. The majority of respondents classified themselves as “White” with other categories making up <4%. This was highly similar to the 2019 sample that was itself representative of the population of Wales (34). The 2020 sample showed a gender imbalance (~80% women) which is not representative of the population. Hence, all statistical analyses were stratified by gender so that any differences due to gender would not affect the results reported. Our sample also showed an under-representation of older adults compared to the 2019 sample.

**Table 1:** Demographic information on the sample and that of the NSfW (34).

		**Number**	**Percent**	**NSfW 2019**
Total		12989		
Gender	Male	2490	19.2	44.9
	Female	10391	80.0	55.1
	Other	25	0.2	–
	Prefer not to say/no response	83	0.6	0.0
Age	16–24	703	5.4	5.8
	25–34	1870	14.4	11.9
	35–44	2647	20.4	13.0
	45–54	3254	25.1	15.6
	55–64	2761	21.3	18.2
	65–74	775	6.0	19.9
	75+	968	7.5	15.6
	Prefer not to say/no response	11	0.1	0.0
Ethnicity	White—any	12553	96.6	96.4
	Asian—any	130	1.0	1.7
	Black—any	16	0.1	0.5
	Mixed—any	110	0.8	0.5
	Other	74	0.6	0.8
	Prefer not to say/no response	106	0.8	0.1
Relationship status	Single	1847	14.2	28.4
	Married/civil partnership	7101	54.7	45.2
	Co-habiting	1880	14.5	–
	Partner non-cohabiting	753	14.2	–
	Separated	198	1.5	2.4
	Divorced	652	5.0	11.8
	Widowed	406	3.1	12.2
	Other	69	0.5	–
	Prefer not to say/no response	83	0.6	0.1
Employment	Paid employment	8533	65.7	46.3
	Self-employed	502	3.9	
	Student	480	3.7	3.7
	Apprentice	31	0.2	–
	Unemployed	149	1.1	2.1
	Long term sick/disability	413	3.2	5.5
	Retired	1945	15.0	36.6
	Furloughed	574	4.4	–
	Stay at home parent	228	1.8	4.7
	Full time carer	42	0.3	
	Other	2	0.0	0.8
	Prefer not to say/no response	90	0.7	0.0

### Well-Being Index

**Figure 1:**
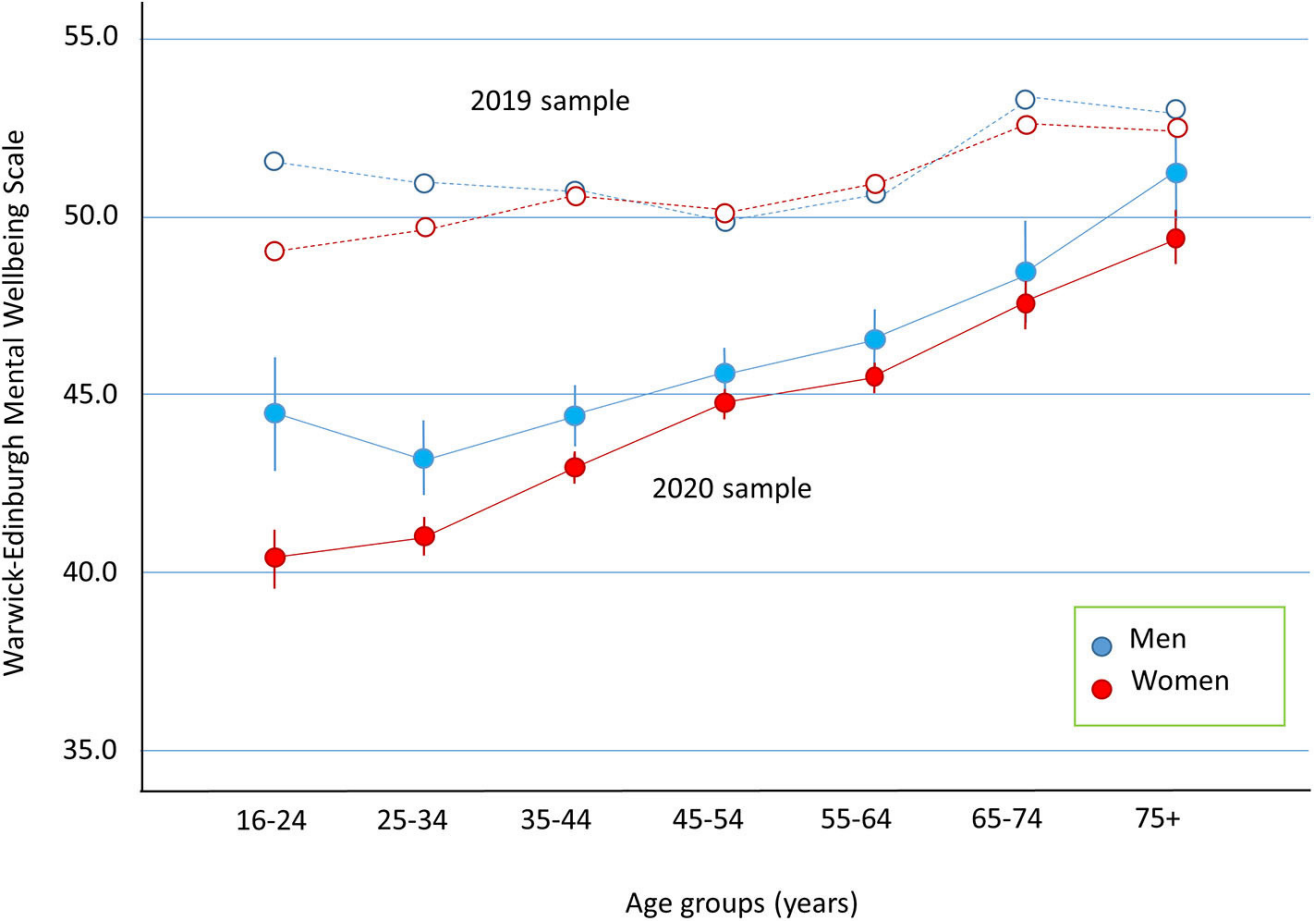
Well-being (WEMWBS) is plotted as a function of age split into 10 year age groups. Error bars represent 95% confidence intervals. Closed symbols are for the 2020 sample and data from the 2019 sample are plotted as open symbols.

Data from the 2020 sample (*n* = 12,554), stratified by gender and age-group, are displayed in Figure 1 (filled symbols). An analysis of variance (ANOVA) showed a small effect of gender, *F*_(1, 12,540)_ = 46.45, *p* < 0.001, η_*p*_^2^ = 0.004, such that men reported higher well-being scores than women (*M* = 46.3 [95% CI: 45.8, 46.7] vs. 44.5 [44.3, 44.8]). There was a main effect of age, *F*_(6, 12,540)_ = 68.12, *p* < 0.001, η_*p*_^2^ = 0.032, such that well-being increased with increasing age (e.g., 16–24 year olds *M* = 42.4 [41.5, 43.3]; 75+ year olds *M* = 50.3 [49.6, 51.0]). The interaction between gender and age was not significant.

Data from the 2019 sample (*n* = 9,753) are also plotted in Figure 1 (open symbols). Scores for the 2020 sample were significantly lower than for the 2019 sample, *F*_(1, 22,279)_ = 1,215.12, *p* < 0.001; η_*p*_^2^ = 0.05 (M = 45.4 [45.2, 45.6] vs. *M* = 51.1 [50.9, 51.3]). The data from the 2019 sample were taken across a year-long period, whereas the 2020 sample were taken in the months of June and July. To account for possible time of year effects, we examined the 2019 data by month. In the months of June and July the mean WEMWBS was 51.4 [50.7, 52.0] which was slightly above the mean for the year (*M* = 51.2 [51.1, 51.4]). Hence, well-being was higher in the months of June and July for the 2019 sample, and so time of year cannot account for the present findings of low psychological well-being in the 2020 sample.

These overall differences between the samples were moderated by interactions with both gender, *F*_(1, 22,279)_ = 10.58, *p* = 0.001; η_*p*_^2^ = 0.001, and age, *F*_(1, 22,279)_ = 21.60, *p* < 0.001; η_*p*_^2^ = 0.006. The 3-way interaction term was not significant.

The interaction with gender is detailed in Table 2. The WEMWBS dropped by a greater amount from the 2019 sample to the 2020 sample for women than for men, although this effect size is small.

**Table 2:** Results from the Warwick-Edinburgh Mental Wellbeing Scale. Numbers in square brackets represent 95% confidence intervals.

		**Warwick Edinburgh Mental Well-Being Score**		**Difference [95% CI]**	***p***	**Effect size (Hedges G) [95% CI]**	**Low mental health/probable depression (WEMWBS** **≤** **40)**		**Odds** **ratio**
Sample		2020	2019				2020	2019	
Gender	Male	45.9	51.5	5.6 [5.1, 6.1]	<0.001	0.57 [0.52, 0.62]	30.0 [28.2, 31.8]	12.0 [11.1, 13.0]	3.14 [2.76, 3.56]
	Female	44.2	51.0	6.8 [6.4, 7.1]	<0.001	0.70 [0.66, 0.73]	35.5 [34.6, 36.4]	12.4 [11.6, 13.3]	3.94 [3.59, 4.31]
Age	16–24	41.2	50.3	9.1 [8.0, 10.2]	<0.001	0.95 [0.83, 1.06]	46.1 [42.3, 49.9]	14.0 [11.3, 17.0]	7.20 [5.45, 9.50]
	25–34	41.4	50.2	8.8 [8.1, 9.4]	<0.001	0.92 [0.84, 1.00]	47.5 [45.2, 49.8]	14.9 [12.9, 17.0]	6.31 [5.26, 7.57]
	35–44	43.2	50.7	7.5 [6.8, 8.1]	<0.001	0.79 [0.72, 0.86]	38.3 [36.5, 40.3]	13.7 [11.9, 15.7]	3.91 [3.28, 4.67]
	45–54	44.9	50.1	5.1 [4.6, 5.7]	<0.001	0.53 [0.47, 0.59]	32.4 [30.7, 34.0]	15.3 [13.5, 17.1]	2.66 [2.27, 3.10]
	55–64	45.7	50.8	5.1 [4.6, 5.7]	<0.001	0.51 [0.45, 0.57]	30.3 [28.6, 32.1]	13.7 [12.1, 15.3]	2.75 [2.35, 3.22]
	65–74	47.8	52.9	5.1 [4.4, 5.9]	<0.001	0.56 [0.47, 0.65]	23.3 [20.3, 26.4]	8.0[6.9, 9.4]	3.48 [2.75, 4.41]
	75+	49.8	52.7	2.9 [2.1, 3.7]	<0.001	0.31 [0.22, 0.39]	16.4 [14.1, 19.0]	8.2[6.8, 9.8]	2.21 [1.70, 2.88]

The interaction with age is also detailed in Table 2. The change in scores from the 2019 sample to the 2020 sample were systematically smaller as a function of increasing age group. Hence, the youngest age group has a difference of 9.1 points, while the oldest had a difference of 2.9 points.

**Figure 2:**
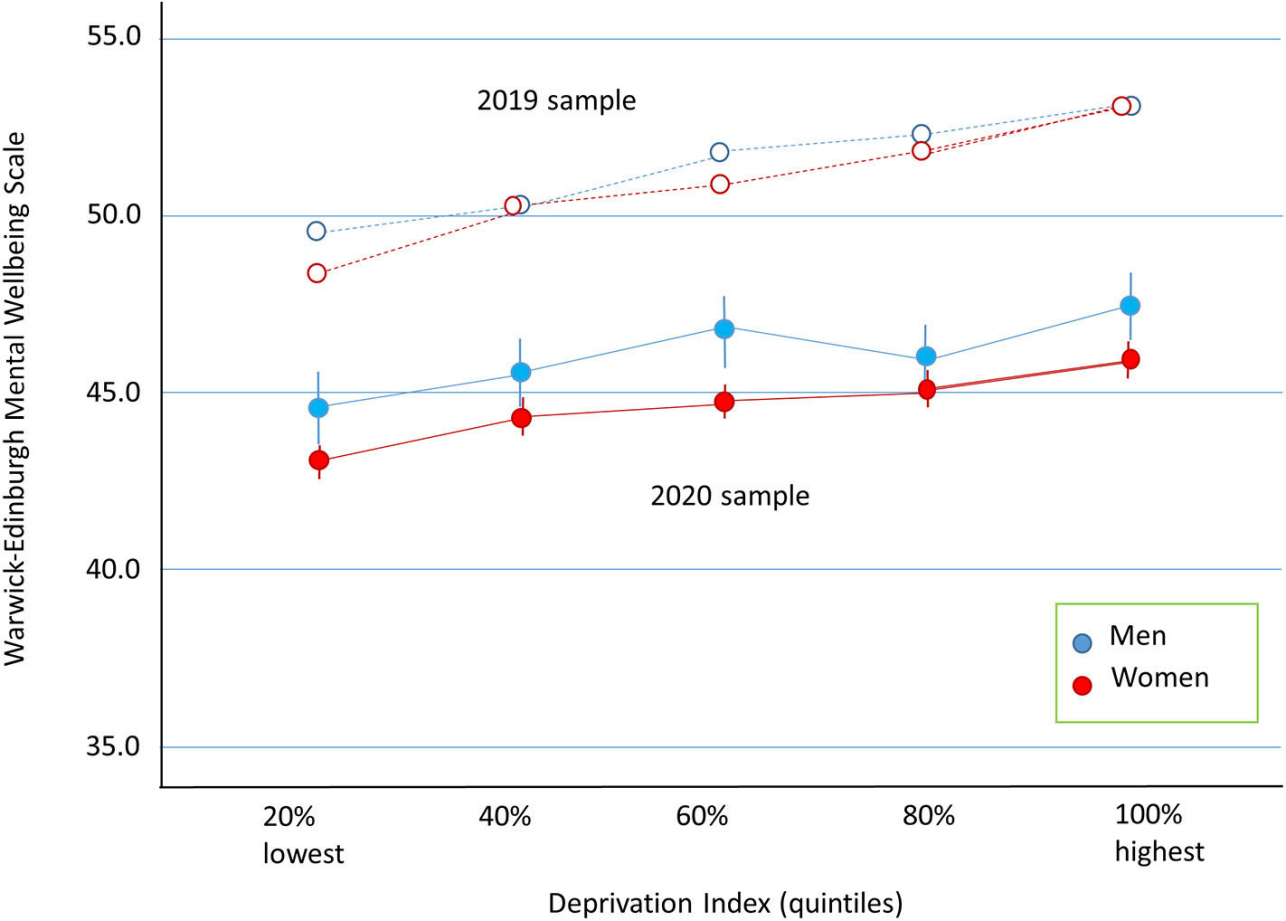
Well-being (WEMWBS) is plotted as a function of WIMD split into quintiles. Error bars represent 95% confidence intervals. Closed symbols are for the 2020 sample and data from the 2019 sample are plotted as open symbols.

Well-being as a function of deprivation index (*n* = 9,726) is displayed in Figure 2 for the 2020 sample. ANOVA showed a small effect of gender, *F*_(1, 9, 716)_ = 32.57, *p* < 0.001, η_*p*_^2^ = 0.003, and of deprivation index, *F*_(4, 9, 716)_ = 14.17, *p* < 0.001, η_*p*_^2^ = 0.006, but the interaction was not significant. The results show that psychological well-being was reduced with increasing levels of deprivation.

### Psychological Distress

The K10 was included in the study because of its well-established ability to categorize people in terms of clinically significant levels of mental distress. Scores on the K10 were used to categorize people into “psychologically well (0–19),” “mild mental disorder/distress (20–24),” “moderate mental disorder/distress (25–29),” and “severe mental disorder/distress (30+).”

Using these criteria, 56.4% of the total sample (*n* = 12,415) showed clinically significant levels of mental distress (see Table 3 for the full set of results). Further, in the present sample, 20.2% reached the criteria for “severe mental distress.”

**Table 3:** Results from the K10 measure of mental distress. Numbers in square brackets represent 95% confidence intervals.

**K10**		**Score**	**Well (%)**	**Mild (%)**	**Moderate (%)**	**Severe (%)**	**Odds ratio (severe)**
All		22.2 [22.1, 22.4]	43.6 [42.7, 44.4]	19.6 [18.9, 20.3]	16.7 [16.0, 17.4]	20.2 [19.5, 20.9]	–
Gender	Male	20.8 [20.5, 21.1]	52.6 [50.6, 54.6]	17.6 [16.1, 19.2]	12.8 [11.5, 14.2]	17.0 [15.6, 18.6]	1.00
	Female	22.6 [22.4, 22.8]	41.4 [40.4, 42.4]	20.1 [19.3, 20.9]	17.6 [16.9, 18.4]	20.9 [20.1, 21.7]	1.29 [1.14, 1.44]
Age	16–24	26.5 [25.9, 27.2]	23.4 [20.2, 26.6]	20.1 [17.1, 23.2]	20.3 [17.2, 23.3]	36.2 [32.6, 40.0]	6.50 [4.90, 8.63]
	25–34	25.4 [25.0, 25.8]	28.4 [26.4, 30.6]	19.4 [17.6, 21.3]	20.0 [18.2, 22.0]	32.2 [30.0, 34.4]	5.30 [4.15, 6.93]
	35–44	23.0 [22.7, 23.4]	38.3 [36.3, 40.1]	21.7 [20.1,23.4]	19.2 [17.7, 20.8]	20.9 [19.3, 22.5]	2.97 [2.31, 3.84]
	45–54	21.8 [21.5, 22.0]	45.8 [44.0, 47.5]	20.3 [18.9, 21.7]	15.7 [14.5, 17.1]	18.2 [16.9, 19.6]	2.51 [1.95, 3.23]
	55–64	21.1 [20.8, 21.4]	49.9 [48.0, 51.9]	18.1 [16.7, 19.6]	15.6 [14.2, 17.0]	16.4 [15.0, 17.8]	2.21 [1.71, 2.86]
	65–74	19.5 [18.9, 20.0]	57.3 [53.7, 60.1]	19.3 [16.5, 22.3]	12.8 [10.5, 15.4]	10.6 [8.5, 13.1]	1.34 [0.96, 1.87]
	75+	18.2 [17.7, 18.6]	66.2 [63.1, 69.4]	15.7 [13.3, 18.0]	10.0 [8.1, 12.1]	8.0[6.2, 9.8]	1.00
Deprivation Index	1 (most deprived)	23.2 [22.8, 23.6]	39.8 [37.6, 42.1]	19.6 [17.8, 21.4]	16.2 [14.6, 18.0]	24.4 [22.5, 26.3]	2.05 [1.74, 2.42]
	2	22.2 [21.9, 22.6]	42.8 [40.6, 45.1]	21.5 [19.7, 23.4]	16.1 [14.5, 17.8]	19.5 [17.8, 21.4]	1.54 [1.29, 1.82]
	3	21.9 [21.5, 22.2]	45.2 [43.0, 47.5]	18.9 [17.2, 20.7]	17.0 [15.4, 18.8]	18.9 [17.2, 20.8]	1.49 [1.26, 1.78]
	4	21.7 [21.3, 22.0]	47.2 [45.0, 49.5]	18.0 [16.3, 19.8]	16.9 [15.3, 18.7]	17.9 [16.2, 19.7]	1.39 [1.17, 1.65]
	5 (least deprived)	20.5 [20.2, 20.9]	52.2 [49.9, 54.4]	20.2 [18.4, 22.0]	14.4 [12.9, 16.1]	13.8 [12.2, 15.4]	1.00

Age was associated with psychological distress, χ^2^(18, *N* = 12,407) = 762.37, *p* < 0.001, see Table 3. Levels of distress were least in the oldest (75+) group, with 33.8% having clinical significance and 8.0% being classified as “severe,” and greatest in the youngest group (16–24) with 76.6 and 36.2%, respectively. Calculation of odds ratios using the oldest group as the comparison shows that individuals with severe psychological distress are 6.50 times more likely to be in the youngest age group in comparison to the oldest age group.

The extent of deprivation also influenced the proportions classified as mentally unwell on the K10, χ^2^(12, *N* = 9,629) = 107.56, *p* < 0.001. Details are shown in Table 3. To illustrate, levels of “severe” psychological distress were greatest in the most deprived group (24.4%) and were nearly double that of the least deprived group (13.8%). Using the least deprived group as a comparison, individuals with severe psychological distress were 2.05 times more likely to be in the most deprived group compared to the least deprived group.

## Discussion

The data show lower levels of mental well-being during the COVID-19 pandemic (2020 sample) as compared to data collected in the year prior to the COVID-19 pandemic (2019 sample). In turn, the data show high levels of psychological distress during the COVID-19 pandemic, with around 50% of the population reporting clinically significant levels of psychological distress, and around 20% showing “severe” effects. This was particularly apparent in younger people, where around 1/3 of individuals are reporting “severe” levels of psychological distress. Psychological distress is also higher in women and those from deprived areas. These findings are broadly in line with previous studies (6, 8, 11, 12) but represent a more extreme effect.

### Effects of Age

The finding of a greater effect of the pandemic on younger adults may be viewed as surprising given that COVID-19 causes far more serious illness and has greater lethality as a function of increasing age (45). There have also been reports of far greater anxiety due to COVID-19 in older adults in the UK (46). However, similar findings that the mental health of young adults has been most affected have been reported in previous studies published on this topic to date (6, 8, 11, 12).

Any stressor that affects the whole population will produce more people entering the “severe” category for those groups that already have lower well-being scores before the stressor, *via* a simple “additive” model. Levels of psychological distress using the K10 have been shown to reduce with age in other populations (47), while well-being is less affected by age (28, 48). However, the present data comparing scores on the WEMWBS in the year prior to the onset of the COVID-19 pandemic and during COVID-19 suggest an interaction whereby the stressors due to COVID-19 have a greater effect on younger people in the population (a non-additive model) producing an even greater number of young people who fall into the “severe” category compared to what would be predicted from a simple additive model.

The reasons for the greater effect of COVID-19 on the mental health of younger adults are not known. It is known that frequent social interaction outside of the immediate family, and forming and maintaining friendships, may be particularly important at this age and their loss more stressful or difficult to tolerate psychologically. For example, Roach (49) provides a review of the importance of peer relationships for mental health in adolescents and concludes that such relationships are protective against anxiety, depression, and suicidal ideation. Further, Beam and Kim (50) note that social isolation caused by the COVID-19 pandemic may affect young adults more than other age groups. Alternatively, older adults might have less stress due to such factors as financial security or employment stability (or retirement, etc.). Further research is needed to be able to isolate the “active” elements of the COVID-19 pandemic, and the corresponding community lock-down, upon deteriorating mental health so that public health interventions designed to ameliorate psychological distress on a population-wide level can be used to target the most potent factors.

### Effects of Gender

Our data indicate greater levels of mental health problems due to COVID-19 in women and these results are consistent with previous findings (6, 8, 11, 12). Pre-COVID-19 studies have also indicated greater levels of psychological distress in women of a similar magnitude (51). While the reasons underpinning these gender differences are unclear, it is important to interpret these findings within the context of the robust gender differences in stress and coping (52, 53) and the gender differences in personality traits that may underpin these effects (54). The resulting picture is that the number of women requiring mental health support and intervention due to COVID-19 is likely to be greater than that for men and the possibility that there may be a need for the development of different intervention strategies depending on gender.

### Effects of Deprivation

The finding that economic deprivation has a negative effect on well-being and mental health is well-documented [e.g.,WEMWBS see (55); K10 see (51)]. While the data are clear in showing higher levels of psychological distress as a function of deprivation index, it is not clear from our data if this is merely due to a lower overall level of mental health pre-COVID-19 (an additive effect) or whether the COVID-19 pandemic has had a greater overall impact on people from more deprived areas. Either way, the implication of these results is that mental health and support services that cover areas of higher deprivation are likely to see a greater demand for psychological and mental health intervention and that communities and governments will need to plan for this increase in population need.

Finally, it should be noted that our index of deprivation was indirect as it was based on the participant's postcode. Future research may wish to gather more direct data about the individual circumstances of the person.

### Limitations and Future Research

The current data has several limitations. First, the 2020 sample was recruited from adults living across Wales and appears to reflect the demographics of this population. However, our recruitment strategy meant that certain sectors of the population who might be more at risk of experiencing psychological distress or negative impacts on mental well-being were not sampled in sufficient detail for us to be able to give separate estimates of well-being and psychological distress for these groups. For example, Black Asian and Minority Ethnic groups (BAME) in the UK appear to have suffered greater mortality rates due to COVID-19 than the white ethnic population (56), and this could well be reflected in a greater deterioration, or a greater impact of COVID-19, on mental health and well-being in BAME communities.

Second, the present study did not sample from people aged 15 or lower. Given that the data show the greatest impact on psychological distress in the youngest age group sampled, data are needed on these young people and on children and adolescents living through the COVID-19 pandemic so that appropriate intervention strategies can be applied, if necessary.

Third, the survey technique (online collection with snowballing advertisement) for the 2020 sample differs from the 2019 sample where face-to-face interviews were conducted on selected households to represent the population of Wales. Importantly, the demographic data from the 2020 sample appears to be in close accord with that of the 2019 sample. The exception to this is that our sample contained fewer people in the older age groups. The reason for this is not known, but it seems probable that this reflects less usage of social media and access to the internet. However, this leaves open the possibility that only the more psychologically healthy older adults completed the survey. If so, then our figures may represent an underestimation of mental health issues in the population and in this age group.

Fourth, the 2020 and 2019 samples may differ on other, non-measured, factors. The most obvious of these factors is the willingness of an individual to complete such a survey, which may be biased toward those people who have been most affected by the COVID-19 pandemic or who have more interest in mental health issues. However, this limitation is inherent to all survey techniques (8), although whether the effect is greater in the present sample is not known.

Finally, this research aimed to examine how the COVID-19 pandemic affected the mental health and well-being of the Welsh population across key demographic variables (age, gender, socioeconomic status). This research cannot provide more specific information on how potentially psychologically vulnerable subgroups have been affected by the COVID-19 pandemic. Certain subgroups such as individuals with specific mental health diagnoses (57), individuals who experienced childhood maltreatment (58), or individuals with abnormal sensory processing patterns (59) are more vulnerable to negative psychiatric and mental health outcomes and future research should investigate how these groups have been affected by the COVID-19 pandemic.

### Conclusion

The data point to a decrease in psychological well-being in the people of Wales in the period 11–16 weeks since the implementation of lockdown measures due to the COVID-19 pandemic. In turn, this translates to an increase in clinically significant levels of psychological distress in Wales, with a 3- to 4-fold increase in those classed as having “severe” problems. The problems appear to be particularly severe in younger adults and also greater for women, and for those from areas of greater deprivation. These important findings can be used to prepare and plan for the wave of psychological distress that has been predicted to hit mental health and support services due to the COVID-19 pandemic and which now appear to be materializing. Given the consistency of our findings with data from the USA (6) and the UK (8, 11), we suspect that similar patterns of deteriorating mental health will emerge in other countries. We are learning that the impact of the pandemic itself, and the emergency governmental response to it, have not only had profound economic consequences, but have also had a significant impact on the mental health of the Nation.

### Added Value of This Study

This is the first study to examine mental health of a nation for a period well into the COVID-19 crisis and lock-down (11–16 weeks) and to compare this to data for a comparable sample before the advent of COVID-19 (2019). We also took measures of both psychological well-being and of clinically significant levels of mental distress using well-established instruments. We found levels of poor psychological well-being and mental distress that were well above pre-COVID-19 levels and far greater than the previous studies of the early period of the crisis. We found these problems were not evenly distributed across the population, but had a more dramatic effect on younger adults. Greater levels of mental distress were also found in women and those from the most deprived areas, although these effects were more modest.

### Implications of All the Available Evidence

The data point to a dramatic decrease in the mental health of the nation of Wales, with over 20% of people reporting “severe” levels of distress. While the physical effects of COVID-19 might be most apparent in older adults, the effects on mental health are more severe in younger people. The data point to the need for government and local health services to prepare for a wave of mental health problems which may follow in the footprints of the pandemic. While the active ingredients causing the mental health deterioration have not yet been isolated, there is a need to balance the efforts to stop the spread of the virus against the mental health problems being caused by the crisis. Our data, compared to that of studies published earlier in the COVID-19 pandemic, point to a deepening problem that is likely to continue with possible “second waves” of infection and the effects of economic problems precipitated by the pandemic and governmental response to it. Continued monitoring of the situation is required, alongside studies that examine which aspects of the pandemic are responsible for this deterioration of the mental health of a nation.

## Data Availability Statement

The raw data supporting the conclusions of this article will be made available by the authors, without undue reservation.

## Ethics Statement

The studies involving human participants were reviewed and approved by Research Ethics Committee at the College of Health Sciences, Swansea University. Written informed consent from the participants' legal guardian/next of kin was not required to participate in this study in accordance with the national legislation and the institutional requirements.

## Author Contributions

CO'C and NG devised the study concept. JK and NG wrote the research proposal and sought and obtained ethical approval and research governance permissions for the research from each of the seven Health Boards in Wales. CO'C coordinated the Health Boards' response to the research. JP and NS created the online survey in Qualtrics, monitored and downloaded data, and wrote scripts for data translation and analysis. NS wrote the Welsh translations. SW designed and implemented the website and led on social media engagement. NG, JK, NS, and RS wrote the study registration on ISRCTN and Research Protocol, now published online by Swansea University. RS wrote the initial analysis plan with input from all authors, carried out data analysis, produced figures, and wrote the first draft of the manuscript. JK checked all the analyses. All authors collaboratively devised the survey questions and aims, contributed to the dissemination of the survey, and contributed to editing and commenting on the final version.

## Conflict of Interest

The authors declare that the research was conducted in the absence of any commercial or financial relationships that could be construed as a potential conflict of interest.
